# Correction: Aromaticity and sterics control whether a cationic olefin radical is resistant to disproportionation

**DOI:** 10.1039/d1sc90142g

**Published:** 2021-06-29

**Authors:** Julian Messelberger, Annette Grünwald, Stephen J. Goodner, Florian Zeilinger, Piermaria Pinter, Matthias E. Miehlich, Frank W. Heinemann, Max M. Hansmann, Dominik Munz

**Affiliations:** Lehrstuhl für Allgemeine und Anorganische Chemie, Friedrich-Alexander-Universität Erlangen-Nürnberg Egerlandstr. 1 91058 Erlangen Germany dominik.munz@fau.de; Institut für Organische und Biomolekulare Chemie, Georg-August Universität Göttingen Tammannstraße 2 37077 Göttingen Germany; Organische Chemie, Technische Universität Dortmund Otto-Hahn-Str. 6 44227 Dortmund Germany

## Abstract

Correction for ‘Aromaticity and sterics control whether a cationic olefin radical is resistant to disproportionation’ by Julian Messelberger *et al.*, *Chem. Sci.*, 2020, **11**, 4138–4149, DOI: 10.1039/D0SC00699H.

The authors regret that funding details were incorrect in the acknowledgements section of the original article. The corrected acknowledgements section for this article is shown below.

 

**Acknowledgements**

D. M. and M. M. H. thank the Fonds der Chemischen Industrie for Liebig fellowships. Financial Support by Boehringer Ingelheim Stiftung and the German-American Fulbright commission are gratefully acknowledged. We thank the RRZE Erlangen for computational resources. Continuous support by K. Meyer and M. Alcarazo is gratefully acknowledged.

 

The Royal Society of Chemistry apologises for these errors and any consequent inconvenience to authors and readers.

